# *In silico* Identification of Novel Toxin Homologs and Associated Mobile Genetic Elements in *Clostridium perfringens*

**DOI:** 10.3390/pathogens8010016

**Published:** 2019-01-29

**Authors:** Jake A. Lacey, Priscilla A. Johanesen, Dena Lyras, Robert J. Moore

**Affiliations:** 1 Infection and Immunity Program, Monash Biomedicine Discovery Institute and Department of Microbiology, Monash University, Clayton, Victoria 2800, Australia; Jake.lacey@unimelb.edu.au (J.A.L.); priscilla.johanesen@monash.edu (P.A.J.); dena.lyras@monash.edu (D.L.); 2 Department of Doherty, The Peter Doherty Institute for Infection and Immunology, University of Melbourne, Victoria 3000, Australia; 3 School of Science, RMIT University, Bundoora, Victoria 3083, Australia

**Keywords:** toxin, plasmid, pCW3, pCP13, *Clostridium perfringens*, leukotoxin, epsilon, binary toxin, hemolysin

## Abstract

*Clostridium perfringens* causes a wide range of diseases in a variety of hosts, due to the production of a diverse set of toxins and extracellular enzymes. The *C. perfringens* toxins play an important role in pathogenesis, such that the presence and absence of the toxins is used as a typing scheme for the species. In recent years, several new toxins have been discovered that have been shown to be essential or highly correlated to diseases; these include binary enterotoxin (BecAB), NetB and NetF. In the current study, genome sequence analysis of *C. perfringens* isolates from diverse sources revealed several putative novel toxin homologs, some of which appeared to be associated with potential mobile genetic elements, including transposons and plasmids. Four novel toxin homologs encoding proteins related to the pore-forming Leukocidin/Hemolysin family were found in type A and G isolates. Two novel toxin homologs encoding proteins related to the epsilon aerolysin-like toxin family were identified in Type A and F isolates from humans, contaminated food and turkeys. A novel set of proteins related to clostridial binary toxins was also identified. While phenotypic characterisation is required before any of these homologs can be established as functional toxins, the *in silico* identification of these novel homologs on mobile genetic elements suggests the potential toxin reservoir of *C. perfringens* may be much larger than previously thought.

## 1. Introduction

*Clostridium perfringens* is a pathogen of humans and animals and is responsible for a wide range of enterotoxigenic and histotoxic diseases that vary in both symptoms and severity. The disease capability of particular strains is due to the production of toxins and extracellular enzymes with specialised roles in pathogenesis. The presence and absence of six major toxins is used to classify *C. perfringens* isolates into seven different toxin types, A-G (Table 1) [1]. Toxin typing is used as an indicator of disease-causing capability as some toxins are strongly associated with disease in certain animal hosts, such as NetB (type G) and necrotic enteritis in chickens, and enterotoxin (type F) in food poisoning. However, toxin typing does not account for the full toxin repertoire a strain may be capable of producing and therefore lacks the high resolution afforded with whole genome sequencing (WGS). Furthermore, the clostridial toxin typing system does not account for strain clonality and is inappropriate for inference of evolutionary relationships [2] as many of the toxins are encoded on large plasmids [3] and capable of horizontal gene transfer [4,5,6].

Excluding the 6 genes used in toxin typing strains, a further 16 toxins and enzymes have been described in *C. perfringens* including sialidases (NanI, NanJ, NanK), hyaluronidases (NagH, NagI, NagJ, NagK)*,* collagenase (Kappa), Beta2 (consensus and atypical variants), TpeL, Delta, BecAB/CPILE and NetE, NetF, NetG. Many of these toxins are recent discoveries, such as NetB, NetE, NetF, NetG, BecAB, further demonstrating the importance of host-specific toxins which lie outside of the currently defined mechanisms of disease and subsequent toxin typing framework [7,8,9,10].

Pore-forming toxins are commonly associated with disease in *C. perfringens*. The pore-forming toxins are comprised of a single protein that forms multimeric complexes. Each type of pore-forming toxin has distinct domain structures. Six beta-barrel pore-forming toxins have been characterised in *C. perfringens* including beta toxin, delta toxin, NetB, NetE, NetF and NetG [10,11,12,13]. Each of these proteins has a unique amino acid sequence with a shared domain structure which includes a signal sequence followed by a leukotoxin/hemolysin domain. This domain is shared with toxins from other species including *Staphylococcus, Bacillus,* and other *Clostridium* members [10,11,12,13].

The remaining known pore-forming toxins, including alpha toxin (phospholipase C), perfringinolysin O (theta), epsilon toxin and enterotoxin, contain distinctly different functional domains [14,15,16]. Phospholipase C is a hemolysin with sphingomyelinase activity and phospholipase activity [17]. Perfringinolysin O is a pore-forming cholesterol-dependent cytolysin [18]. Epsilon toxin and enterotoxin both belong to the aerolysin-like toxin family but are comprised of distinctly different amino acid sequences and protein structures [19]. While phospholipase C and perfringinoylsin O are chromosomally encoded toxins, epsilon and enterotoxin are located on mobile genetic elements [5,6]. 

The binary toxins in *C. perfringens* (iota toxin and binary enterotoxin (BEC)) are usually composed of an enzyme component (Ia) and a binding component (Ib) [20]. Ib binds to a receptor on targeted cells and Ia is translocated into the cytosol of the cells Ia. ADP-ribosylates actin, resulting in cell rounding and death [20]. Another binary toxin with ADP-ribosylation activity in *C. perfringens* is BecAB/CPILE [9,21]. Binary toxins are present in other closely related species such as *Clostridiodies difficile*, *Clostridium spiroforme* and *Clostridium botulinum* [22,23,24], and have been shown to be associated with disease in these bacteria. 

Due to the specificity of many *C. perfringens* isolates to particular animal hosts and disease outcomes, and the diverse pan-genome of the species [4,25], we hypothesize that only a subset of the potential toxins encoded by *C. perfringens* have been identified. The aim of this study was to bioinformatically identify novel virulence-associated genes in previously characterised *C. perfringens* strains to inform and refine future studies, as well as narrow down potential drug or vaccine targets. Here, we describe seven novel protein sequences homologous to known toxins and the associated mobile genetic elements associated with the encoding genes, which were identified from the whole genome sequences (WGS) of a diverse set of previously characterised and described *C. perfringens* isolates. Our study demonstrates the value of reanalysing publicly available WGS data and of collating large WGS datasets for use in comparative genomic analysis.

## 2. Results

### 2.1. Identification of Toxin Homologs in C. perfringens Isolates

To identify toxin homologs, we carried out an investigation of WGS from 240 publicly available *C. perfringens* genomes and short read data (Supplementary Table S1). Short read data was assembled using Spades v3.12.0, assembled genomes were annotated using prokka v1.13.2 using a custom database of *C. perfringens* protein sequences obtained from NCBI. Protein sequences were clustered using Roary v3.12.0. Using a 90% identity threshold each of the *C. perfringens* toxins formed a single protein cluster. Six distinct clusters were observed for the hemolysin/leukotoxin domain toxins: NetB (n = 33), NetE (n = 29), NetF (n = 31), NetG (n = 16), delta (n = 1) and beta toxin (n = 4). Single protein clusters also corresponded to epsilon toxin (n = 2), enterotoxin (n = 92), and two clusters corresponding to the subunits of iota toxin (n = 1). Each of these clusters corresponded to the toxin type of each isolate and demonstrated a high level of conservation within the toxin protein sequence. 

Several additional protein clusters were also annotated as potential virulence factors including four clusters annotated as hemolysin/leukotoxin-like toxins, two clusters annotated as epsilon-like toxins and two clusters annotated as iota-like toxin subunits (Table 2). As the protein identity that was used for assigning protein sequences to a cluster was 90%, each of these seven protein clusters with toxin annotations are distinct protein sequences compared to their toxin homologs, as the sequence divergence is too high for these homologs to be considered as allelic variants. 

To confirm the preliminary annotations of the clusters, representative proteins sequences of each of the toxin homolog clusters were subjected to sequence alignment against the UniProtKB database to investigate the protein domain structures. Four predicted proteins were found to contain leukocidin/hemolysin domains and were accompanied by signal sequences. This general amino acid structure conforms to the *C. perfringens* beta toxin structure. Two predicted proteins were found to contain an ETX/*Bacillus* mosquitocidal toxin MTX2 domain also with signal sequences, with a domain architecture similar to that of epsilon toxin. The two predicted proteins corresponding to the two binary toxin components, A and B, showed highly similar domain structures to iota toxin. Component A contained two PFO3496 domains and a signal sequence, and component B contained a signal peptide, PA14 domain and three Binary toxB/anthrax toxin PA CA-binding domains. 

### 2.2. Novel Protein Sequences with Leukotoxin/Hemolysin Domain

**Figure 1:**
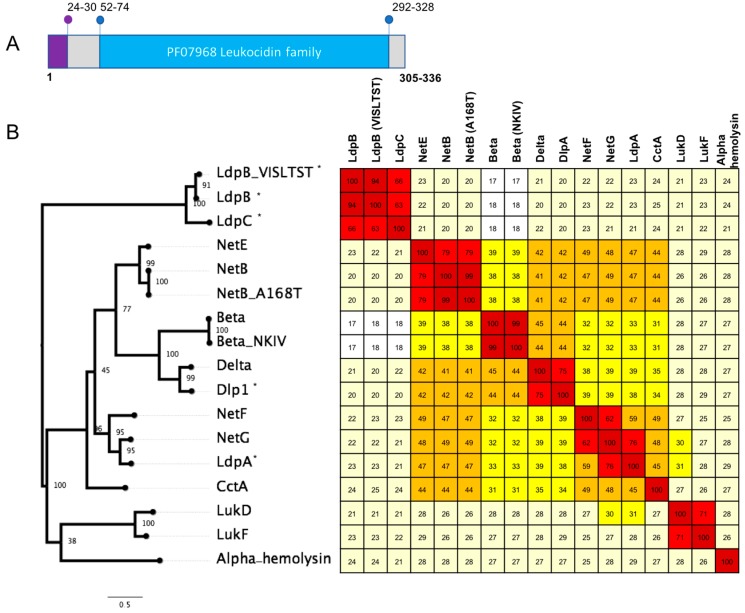
(**A**) Schematic showing key features of the *C. perfringens* leukocidin domain containing proteins. The purple region represents the signal peptide and the blue region the PF07968 leukocidin/hemolysin domain. Numbers marked correspond to amino acid positions of the start and end of the features with the range showing the variation between the different protein sequences (n = 13). (**B**) A maximum likelihood tree based on alignment of novel toxin homologs (marked with *) against protein sequences of the *C. perfringens* leukocidin domain containing proteins and representative sequences of CctA from *Clostridium chauvei* the *Staphylococcus aureus* hemolysin, leukotoxin components F and D. Protein sequences were aligned using clustal omega, and maximum likelihood was implemented in IQtree. The tree was inferred using the LG+F+G4 model and rapid bootstrapping -bb 2000; bootstrap support is shown at the nodes. Scale bar indicates the number of changes per site. Heatmap shows percent identity matrix of protein alignments, colours correspond to the following percent identity: dark red, 80–100%; light red, 60–79%; orange, 40–59%; bright yellow, 30–39%; pale yellow, 20–29% and white, <19%.

Genes encoding four distinct leukocidin domain proteins that have similar structure to the beta-pore forming toxins (beta, delta, NetB, NetE, NetF, NetG toxins) were identified. Based on sequence similarity to the other leukocidin domain proteins in *C. perfringens* and topology of maximum likelihood phylogeny of sequence alignment, we designated these four proteins delta-like protein A (DlpA), leukocidin domain protein A (LdpA), leukocidin domain protein B (LdpB) and leukocidin domain protein C (LdpC) (Figure 1).

**Figure 2:**
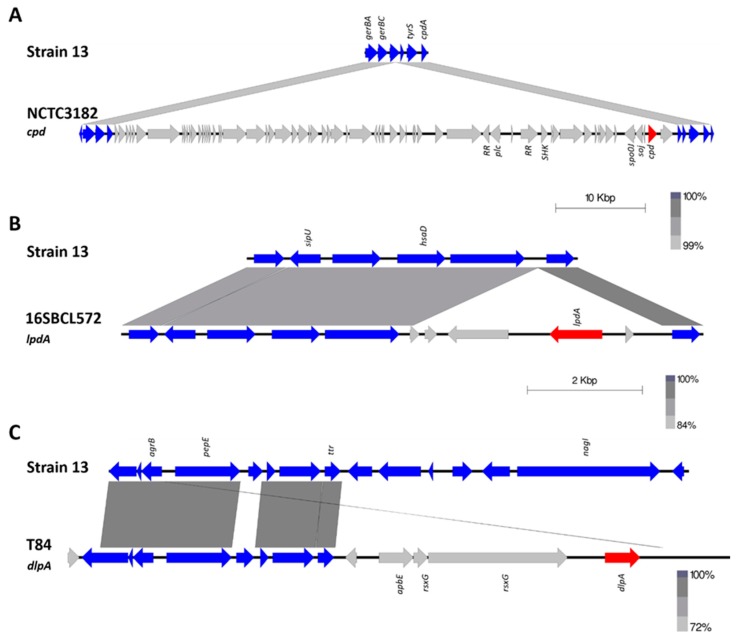
Schematic representation of the genomic location of delta toxin (*cpd*) in NCTC3182 and leukotoxin domain protein A (*ldpA*) from strain 16SBCL572 and delta-like protein (*dlpA*) from strain T84. Chromosomal regions are coloured blue, the unique regions grey, and toxin genes are coloured in red. Genbank accession numbers for sequences are as follows: T84 *dlpA* region (MK285064), NCTC3182 *cpd* region (MK285058), and 16SBCL571 *ldpA* region (MK285056).

Delta-like protein A (DlpA) has 75.31% identity (amino acid) to delta toxin and the next closest sequence is beta toxin, with 44% identity (Figure 1). Three turkey isolates (one from a healthy bird (T43) and two from birds afflicted with necrotic enteritis (T46 and T84)) were found to encode DlpA. DlpA also appears to be chromosomally encoded, however it was not possible to identify the size of the element. Delta toxin was also found to be chromosomally encoded in NCTC3182 (type C) on a large genomic island of ~50 kb size (Figure 2). The presence of both of these toxin genes within otherwise conserved chromosomal regions suggest a chromosomal integration of the delta and DlpA-encoding genes in some isolates. 

The leukotoxin domain protein A (LdpA) was found in two isolates: 16SBCL571 (2015, France, Paris, spices vegetables) and 16SBCL572 (2015, France, Essonne, salad vegetables). Maximum likelihood of sequence alignment and percentage identity shows LdpA is most similar to the NetG and NetF toxins, with 76% and 62% sequence identity, respectively (Figure 1). LdpA is chromosomally encoded and appears to have been incorporated in this location through the insertion of an ~4 kb region. Sequence alignment shows the corresponding genome sites in reference Strain 13 (Figure 2). 

**Figure 3:**
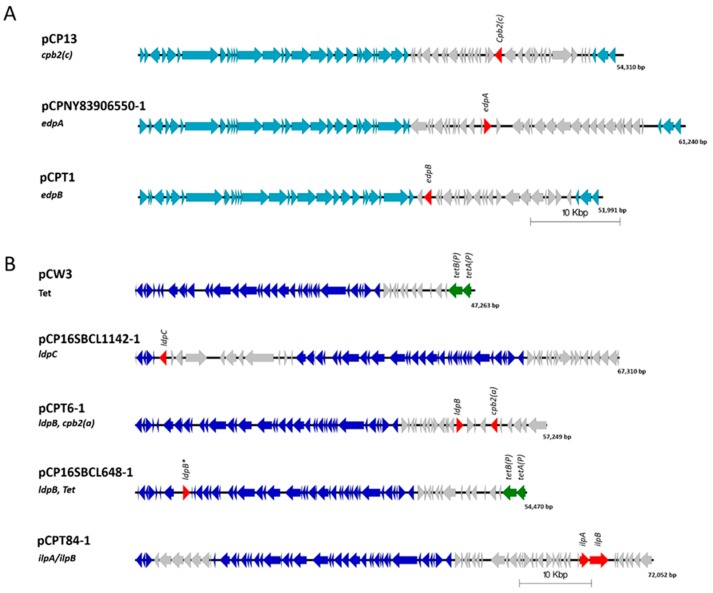
Schematic representation showing the comparative alignment of sequenced *C. perfringens* plasmids compared to plasmid contigs containing toxin homologs, made in EasyFig v2.2.2. (**A**) Shown are a sequence alignment of pCP13 (*cpb2*-con) compared to the epsilon domain protein containing plasmids pCPNY83906550-1 (*edpA*) and pCPT1 (*edpB*). (**B**) Shown are sequence alignments of pCW3 (Tet), pCP16SBCL1142-1 (LdpC), pCPT6-1 (*ldpB*, *cpb2*-atyp), pCP16SBCL648-1 (*ldpB*, Tet) and pCPT84-1 (*ilpA*/*ilpB*). The ORFs in the conserved backbone for pCP13-like plasmid are depicted as light blue arrows. The ORFs in the conserved backbone for pCW3-lke plasmids are depicted as dark blue arrows. Virulence factors and toxin homologs are labelled and other open reading frames are shown as red arrows. Light grey arrows represent open reading frames that are unique to that plasmid, * denotes plasmids containing a toxin homolog. Genbank accession numbers for plasmid sequences are DQ366035 for pCW3, AP003515 for pCP13, MK285071 for pCPNY83906550-1, MK285059 for pCPT1, MK285071 for pCP16SBCL1142-1, MK285060 for pCPT6-1, MK285061 for pCP16SBCL648-1 and MK285057 for pCPT84-1.

The leukotoxin domain protein B (LpdB) was found in four isolates and was comprised of two sequence variants that share 93.62% identity. Three isolates contained a LpdB variant, two from chickens (WER-NE36, EHE-NE7), and one from a turkey afflicted with necrotic enteritis (T6). In these three isolates, LpdB was identified on a pCW3-like plasmid (~57 kb), flanked by two transposons proteins and co-located atypically with *cpb2* and a partial set of genes described as NELoc-2 [26]. The other variant of LpdB was found in isolate SRR7601223 (16SBCL648, unknown source, Paris). It contained seven amino acid substitutions with the majority in the 5’ region of the sequence, in the signal peptide region. This variant was also located on a pCW3-like plasmid but with a significantly different variable region. It was instead co-located with the tetracycline resistance genes *tetA(P), tetB(P)* (Figure 3).

The leukotoxin domain protein C (LdpC) was also found in four isolates. Three isolates from turkeys (two from birds afflicted with necrotic enteritis (T6, T34) and one from a healthy turkey (T53)), and a single isolate from Paris SRR7601202 (16SBCL1142, source unknown, France, 2015). LdpB and LpbC share 63–65.55% identity to each other (Figure 1). LdpC was found to be carried on a pCW3-like plasmid, co-located with a bacteriocin-like locus of an approximate size of 72 kb (Figure 3). While the domain structure of LdpB and LdpC matches what is observed for the other leucocidin/hemolysin domain proteins, the sequence identity ranges from 18.31% to 23.32% in comparison to the previously characterised toxins (Figure 1). These two leukotoxin domain proteins are the most divergent sequences of similar domain proteins in *C. perfringens*.

### 2.3. Novel Protein Sequences with Epsilon Toxin-Like Aerolysin Domain

**Figure 4:**
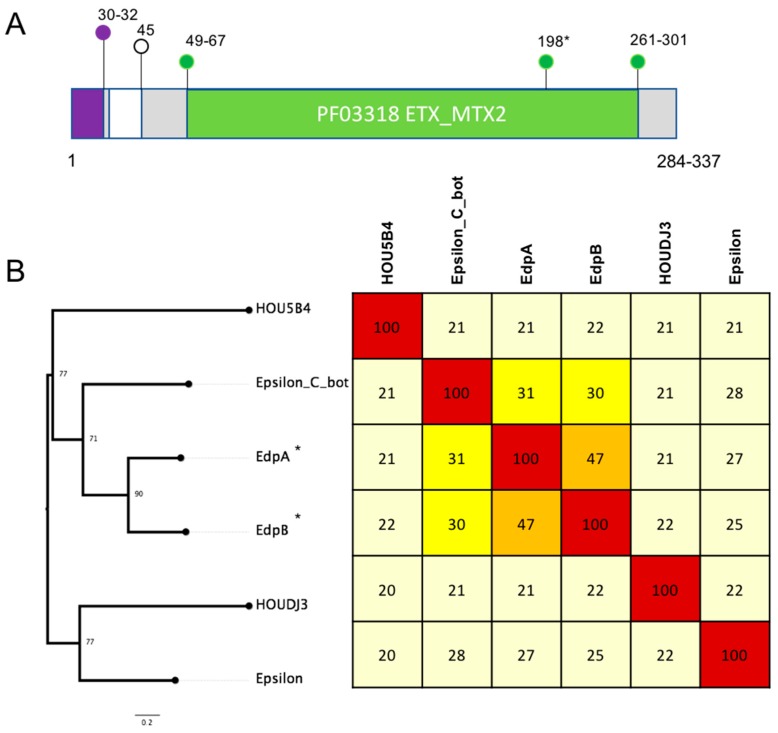
(**A**) Schematic showing key features of the *C. perfringens* epsilon aerolysin domain family proteins, as characterised in Pfam. The PF03318 epsilon toxin ETX/*Bacillus* mosquitocidal toxin MTX2 domain is coloured green, the signal peptide is coloured purple and amino acid positions of the domains are marked. Numbers marked correspond to amino acid positions of the start and end of the features with the range showing the variation between the different protein sequences. (**B**) A maximum likelihood tree based on alignment of novel toxin homologs (marked with *) against protein sequences of the *C. perfringens* epsilon domain containing proteins and representative sequences from *B. lacterosporus* (HOU5B4, HOUDJ3) and epsilon toxin from *C. botulinum*. Protein sequences were aligned using clustal omega, and maximum likelihood was implemented in IQtree. The tree was inferred using the LG+F+G4 model and rapid bootstrapping -bb 2000; bootstrap support is shown at the nodes. Scale bar indicates the number of changes per site. A heatmap showing percent identity matrix of protein alignments is also shown, and colours correspond to the following percent identity: dark red, 80–100%; light red, 60–79%; orange, 40–59%; bright yellow, 30–39%; pale yellow, 20–29% and white, <19%.

Two novel epsilon toxin ETX/Bacillus mosquitocidal toxin MTX2 domain-containing protein sequences were identified (Figure 4). Five isolates contained one of the epsilon homologs designated epsilon domain protein A (EdpA). Two isolates from human blood, specifically, NY83906550 (NY, 2012, human blood) and NY83905249 (human blood, NY, 2010), as well as three isolates from contaminated food, 16SBCL600 (2015, France, poultry sausage), 16SBCL609 (2015, white bean vegetables, France) and 16SBCL1126 (2016, poultry, minced turkey), were found to encode EdpA. The other epsilon homolog designated epsilon domain protein B (EdpB) was found in a single turkey isolate afflicted with necrotic enteritis (T1). 

The novel homologs were compared to the other epsilon domain-containing proteins from *C. perfringens*, *C. botulinum* and *Brevibacillus laterosporus.* Two *C. perfringens* strains, ATCC3626 (type B) and JGS1721 (type D), encode the epsilon toxin with 99.9% nucleotide identity and 99% protein identity between them. Sequence comparison shows that EdpA from all five isolates shared 100% protein identity between them but only 25–27% amino acid identity to the epsilon toxin from ATCC3626 and JGS1721 and 47% to EdpB (Figure 4). EdpA and EdpB shared 30–31% protein identify to the epsilon toxin from *C. botulinum* (Figure 4). 

The novel ETX/MTX2 domain proteins are encoded on pCP13-like plasmids. Both variants encoded the same conserved plasmid regulation regions, but the Turkey isolate T1 has a distinctly different variable region of the plasmid compared to the human isolates. The T1 pCP13-like plasmid is ~52 kb in size while the human isolates carry an ~61 kb plasmid (Figure 3). These plasmids are distinctly different to epsilon toxin-encoding plasmids such as pCP8533*etx*, which have a pCW3-like plasmid backbone. 

### 2.4. Novel Protein Sequences with Similarity to Clostridial Binary Toxins

**Figure 5:**
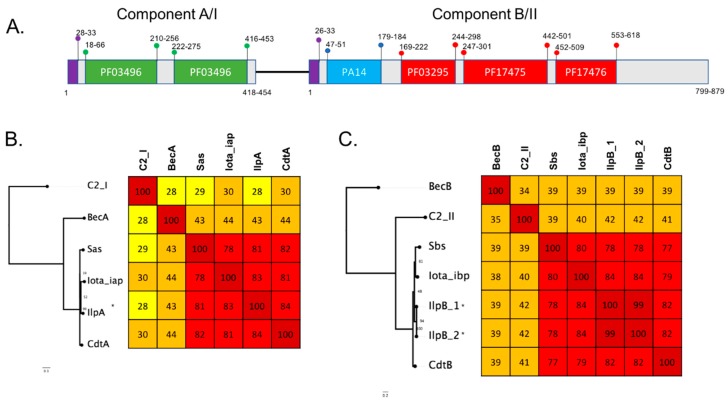
(**A**) Schematic showing key features of the C. perfringens functional domains of the iota binary toxins family of proteins as characterised in Pfam. The PF03496 regions are ADP-ribosytransferase toxin A domains and are coloured green; PA14 is coloured blue; PF03295, PF17475 and PF17476, the toxB domains, are coloured red and the signal peptide is coloured purple. Numbers marked correspond to amino acid positions of the start and end of the features with the range showing the variation between the different protein sequences. (**B**,(**C**)) Maximum likelihood trees based on alignment of novel toxin homologs of each toxin component (marked with *) against protein sequences of the *C. perfringens* binary toxin proteins iota and Bec and representative sequences from *C. botunilim* C2, *C. difficile* binary toxin and *C. spiroforme* binary toxin. Protein sequences were aligned using clustal omega, and maximum likelihood was implemented in IQtree. The tree was inferred using the LG+F+G4 model and rapid bootstrapping -bb 2000; bootstrap support is shown at the nodes. Scale bar indicates the number of changes per site. Heatmap showing percent identity matrix of protein alignments, colours correspond to the following percent identity: dark red, 80–100%; light red, 60–79%; orange, 40–59%; bright yellow, 30–39%; pale yellow, 20–29% and white, <19%.

Four turkey isolates were found to encode homologous protein sequences to the iota binary toxin (IlpA and IlpB). Three of the isolates (T43 healthy bird, T46 diseased bird and T84 diseased bird) were also found to encode DlpA while the fourth (T22 healthy bird) did not. The IlpB from T22 also contained two amino acid substitutions (A226D, R253K) compared to the other three isolates. Sequence comparison of the IlpA and IlpB components show the four turkey isolates have 100% (99% in T22) protein identity to each other, while an 82% protein identity for JGS1987 for the Iap and 84% to Ibp was determined, with most of the sequence vitiation in the 5’ region of the protein. Comparison to other iota-like toxins including BecA and BecB (CPILE) shows an ~43% protein identity to BecA and ~39% identity to BecB (Figure 5). Iota-like toxins are also described in other species and these were compared to the sequences identified in this study. Iota-like toxin from *C. spiroforme* had a 78.4% identity to JGS1987 and 81.72% identify to the putative iota-like toxin. Similar identity was observed to the *C. difficile* binary toxin components CdtA and CdtB. In comparison to phage encoded neurotoxin from *C. botulinum* C2, only 28% identity was observed in C2-I (component A) and 42% in C2-II (component B) (Figure 5). The iota-like sequence was located on a pCW3-like plasmid of ~57 kb in size (Figure 3). Unlike the other toxin homologs characterised in this study, the IlpA/B was found exclusively in turkey isolates.

## 3. Discussion

In this study we identified seven potential toxin homologs with homology to beta, delta, epsilon and iota toxins, and their corresponding putative mobile genetic elements and chromosomal insertions. The toxin homologs were defined based on sequence identity and domain structure of the proteins to the known toxins from *C. perfringens* and other species. The discovery of novel protein sequences with similarity to previously characterised *C. perfringens* toxins suggests that much genetic diversity and toxin diversity still remains to be discovered in this bacterium. The advances in throughput and continuing reduction in the cost of WGS has made it a readily accessible tool for the deeper exploration of bacterial genome structure and content. We demonstrate its use as a screen for putative virulence factors based on domain sequence analysis and a large pool of isolates for which genome data is available. 

Four new leukotoxin domain containing proteins, DlpA, LdpA, LdpB and LdpC, two epsilon domain containing proteins sequences, EdpA and EdpB, and the iota-like protein (IlpAB) were identified in this study. Three of these toxin homologs IlpA/IlpB, DlpA and EdpB, were found exclusively in Type A turkey isolates. Three of the four turkey isolates that carried the plasmid encoded IlpAB also carried DlpA integrated into the chromosome. Two other toxin homologs (LdpB and LdpC) were predominantly identified in isolates from turkeys suffering from necrotic enteritis, but were also identified in isolates from other sources. While there is only a small sample of turkey isolates used in this study (n=13), screening of future isolates from turkeys for these factors may reveal more about the prevalence of these genes and the potential mechanism of virulence in turkeys.

EpdA was found in two isolates from human blood and three different sources of contaminated food, providing a possible association with human disease, although considerably more sampling is required before statistical significance could be reached. It is clear given the diverse geographical range of isolates (France and New York) that the plasmid present in these strains may be widely dispersed. 

While most of the toxin homologs described in this study were found on a single conserved class of plasmid, the LdpB gene was found to be co-located on two different plasmids, a beta2-encoding plasmid in a single turkey isolate and two chicken isolates, as well as on a tetracycline resistance plasmid in an isolate from an unknown source in France. This is a similar observation to enterotoxin and beta2 toxin, which are found to be encoded on multiple different plasmids, however, their co-location with tetracycline resistance is not commonly observed. Both of these plasmids encode a pCW3-like backbone, which shares a common backbone with the IlpAB plasmid. 

The epsilon domain proteins EdpA and EdpB were found to be encoded on a pCP13-like backbone plasmid. The EdpA-encoding plasmid was found intact in five different isolates, while the EdpB-encoding plasmid was found in a single isolate. In contrast, the epsilon toxin is found on a pCW3-like plasmid [5]. These results demonstrate that similar toxins can be found encoded on a diverse range of *C. perfringens* plasmids with different backbones or large variable regions.

Conjugative plasmids play a very important role in *C. perfringens* virulence [3]. A single strain can encode up to four different toxin plasmids, with a single plasmid encoding up to three toxin genes [2,3,7]. *C. perfringens* encodes two different classes of large plasmids, pCP13 and pCW3 [3,13,27], both of which have been demonstrated to be conjugative [27,28,29]. This study has identified six new plasmids, two pCP13-backbone plasmids and four pCW3 backbone plasmids. The location of toxin homologs on conjugative plasmids, sometimes co-localised with other virulence genes and tetracycline resistance, was also observed here. This study therefore provides further support that a significant contribution to the genetic diversity of *C. perfringens* is plasmid mediated and involves unique variable regions, including the toxin homologs, and many other genes are present on each of the plasmids.

Thresholds for protein clustering and annotation of coding sequences are important for pan-genome analysis and identification of putative new proteins. Reducing sequence thresholds too low can result in different toxins being clustered together. For example, reducing thresholds below 85% results in *netB* and *netE* being clustered together, hence the error in a previous study claiming *netB* is present in the dog and horse isolates [25], which has since been corrected as it is clear now that both *netB* and *netE* are established as two different proteins [25]. 

The discovery of four newly identified leukotoxin domain-containing proteins, DlpA, LdpA, LdpB and LdpC, emphasises the diversity of this class of protein in *C. perfringens*. With the recent discovery of NetB, NetE, NetF and NetG [8,10], as well as increased functional work to define the mechanism of action of the toxins, including delta toxin, it has been shown that leukotoxin domain proteins in *C. perfringens* are largely responsible for virulence and pathogenesis in multiple diseases [8,11,30,31] Characterisation of two epsilon domain proteins, and the characterisation of another protein sequence with similarity to the clostridial binary toxins, suggests that there is also a high amount of genetic variability of these toxins classes, and not just within the leukotoxin domains. 

The most widely published method for investigating *C. perfringens* isolates from outbreaks is the use of diagnostic PCR for the toxins used in the typing scheme as well as c*pb2* [1,32,33,34,35,36,37]. This study has shown that toxin diversity may be much greater than previously revealed and restricting diagnostics to PCR may be missing key information regarding *C. perfringens* pathogenesis. We suggest the use of whole genome sequencing for *C. perfringens* diagnostics and virulence investigations, in particular from diverse animal sources, as it can provide a more complete and accurate source of information, particularly on new mechanisms of virulence and associations of genetic elements with particular hosts. 

This analysis has used publicly available genomic data to identify seven novel putative toxin proteins with striking similarities to characterised toxins and has localised the genes of most of them to plasmids. Further investigation, particularly on protein expression and functionality of these proteins in animal hosts or cell lines, is required before conclusions can be drawn about the functionality of these proteins as *C. perfringens* toxins.

## 4. Materials and Methods

The DNA sequences analysed in this study were obtained from two sources: FASTA files of published genomes downloaded from the NCBI genome database and genomes with only unassembled and unannotated sequence reads publicly available were downloaded from the NCBI short-read archive (SRA). Where available, the metadata (disease association, year of isolation, country of isolate) was collected for all genomes. For details of the isolates used in this study refer to Supplementary Table S1. 

For the genomes that required assembly, reads were assembled using Spades v.3.12.0 at default settings. All genomes were annotated with Prokka v1.13.2, and protein clustering was performed using Blastp v2.7.1 and CD-HIT in Roary v3.12.0) [38] with minimum percentage identity of 90% (-I 90) with no splitting of paralogs (-s). Protein sequence was examined for functional domains using the NCBI conserved domains database search and Pfam [39], signal sequences were screened using SignalP 4.1[40]. Sequence homologs were also searched using profile hidden Markov models implemented in HMMER v3.2.1 (hmmer.org) [41]; hmmbuild was used to create a profile for each toxin type (leukotoxin, epsilon and binary toxin) and was created from multiple sequence alignment of protein sequences of each toxin type (leukotoxin, epsilon and binary toxin). The profile was then used to search the pan-genome hmmsearch for proteins sequences with significant matches to each of the toxin profiles (--tblout). 

Maximum likelihood trees of protein sequences based on alignment of novel toxin homologs to representative protein sequences were obtained from NCBI. Protein sequences were aligned using clustal omega [42], and maximum likelihood was implemented in IQtree [43]. The tree was inferred using the LG+F+G4 model, and rapid bootstrapping -bb 2000 and non-parametric bootstrap (-v) [43,44] and visualized in Figtree. Heatmaps were produced using a percent identity matrix based on clustal omega alignments and rounded to the nearest whole number. Heatmap colours correspond to the following percent identity: dark red, 80–100%; light red, 60–79%; orange, 40–59%; bright yellow, 30–39%; pale yellow, 20–29% and white, <19%.

Plasmid assembly was performed *de novo* using Spades v3.12 where reads were assembled. Contigs were scaffolded using the closed plasmids pCW3 and pCP13 as scaffolding references and to assist with gap closure and repeat resolution. Reads were mapped back to contigs for error correction using Pilon [45] and to ensure gap closure of plasmid contigs. Plasmid contigs containing the genes of interest were extracted from the genomes and sequence alignment was performed on plasmid contigs against reference plasmids using tBlastx or blastn to investigate similarity (BLAST 2.7.1+) [46,47,48]. Schematics of alignments were produced using Easyfig v.2.2.2 [49]. Plasmids sequences were deposited to Genbank under the Bioproject accession PRJNA508810; the accession numbers for each plasmid are as follows: MK285071 for pCPNY83906550-1, MK285059 for pCPT1, MK285071 for pCP16SBCL1142-1, MK285060 for pCPT6-1, MK285061 for pCP16SBCL648-1 and MK285057 for pCPT84-1. The accession numbers for chromosomal regions are as follows: MK285064 for T84 *dlpA* region, MK285058 for NCTC3182 *cpd* region, and MK285056 for 16SBCL571 *ldpA* region. Sequences of toxin homologs were deposited to Genbank under the accession numbers: MK285070 for *edpA*, MK285055 for *edpB*, MK285066 for *dlpA*, MK285067 for *ldpA*, MK285068 for *ldpB*, MK285063 for l*dpC*, MK285069 for *ilpA* and MK285065 for *ilpB*.

## 5. Conclusions

Here we have demonstrated through analysis of whole genome sequences a series of novel toxin homologs located on conjugative plasmids and chromosomal insertions in *C*. *perfringens*. Although these genes cannot be assigned as toxins without further molecular and microbiological functional confirmation, the presence of iota, beta, delta and epsilon homologs carried on mobile genetic elements in strains from various backgrounds demonstrates that the plasmid diversity and potential toxin diversity encoded by *C. perfringens* is still widely under-reported.

## Figures and Tables

**Table 1:** The toxin typing scheme of C. perfringens [1].

Toxin Type	Alpha (*plc* or *cpa*)	Beta (*cpb*)	Epsilon (*etx*)	Iota (*iap* and *ibp*)	Enterotoxin (*cpe*)	NetB (*netB*)
**A**	+	-	-	-	-	-
**B**	+	+	+	-	-	-
**C**	+	+	-	-	+/-	-
**D**	+	-	+	-	+/-	-
**E**	+	-	-	+	+/-	-
F *	+	-	-	-	+	-
G *	+	-	-	-	-	+

* type F and G strains were formally categorised as Type A until reclassification in 2018 [1].

**Table 2:** Strains encoding toxin homologs.

Strain	Toxin Type	Host *	Year	Country	Accession	Toxins	Toxin Homologs
T43	A	Turkey, Healthy	2009	Finland	SAMN05933484	*plc*	*dlpA, ilpA/B*
T46	A	Turkey, NE	2010	Finland	SAMN05933485	*plc*	*dlpA, ilpA/B*
T84	A	Turkey, NE	2011	Finland	SAMN05929587	*plc*	*dlpA, ilpA/B*
16SBCL571	A	Contaminated food	2015	France	SAMN09721446	*plc*	*lpdA*
16SBCL572	A	Contaminated food	2015	France	SAMN09721448	*plc*	*lpdA*
WER-NE36	G	Chicken, NE	2010	Australia	SAMN07326176	*plc, netB, cpb2*	*ldpB*
EHE-NE7	G	Chicken, NE	2002	Australia	SAMN07326146	*plc, netB, cpb2*	*ldpB*
T6	A	Turkey, NE	2005	Finland	SAMN05929277	*plc, cpb2*	*ldpB, lpdC*
16SBCL648	A	-	2016	France	SAMN09721463	*plc*	*ldpB*
T34	A	Turkey, NE	2009	Finland	SAMN05933483	*plc*	*ldpC*
T53	G	Turkey, Healthy	2010	Finland	SAMN05929586	*plc, netb,*	*ldpC*
16SBCL1142	A	-	2015	France	SAMN09721467	*plc*	*ldpC*
T22	A	Turkey, Healthy	2009	Finland	SAMN05929282	*plc*	*ilpA/B*
NY83906550	A	Human, Blood	2012	USA	SAMN08466960	*plc, cpb2*	*edpA*
NY83905249	A	Human, Blood	2010	USA	SAMN08466959	*plc, cpb2*	*edpA*
16SBCL600	A	Contaminated food	2015	France	SAMN09721470	*plc*	*edpA*
16SBCL609	A	Contaminated food	2015	France	SAMN09721433	*plc*	*edpA*
16SBCL1126	F	Contaminated food	2015	France	SAMN09721434	*plc, cpe*	*edpA*
T1	A	Turkey, NE	1998	Finland	SAMN05928332	*plc, cpb2*	*edpB*

* NE = Necrotic enteritis; - = unknown.

## Supplementary Materials

The following are available online at http://www.mdpi.com/2076-0817/8/1/16/s1, Table S1: Strains used in study.
